# p53 Acetylation: Regulation and Consequences

**DOI:** 10.3390/cancers7010030

**Published:** 2014-12-23

**Authors:** Sara M. Reed, Dawn E. Quelle

**Affiliations:** 1 Department of Pharmacology, The University of Iowa Carver College of Medicine, Iowa City, IA 52242, USA; E-Mail: sara-reed@uiowa.edu; 2 Medical Scientist Training Program, The University of Iowa Carver College of Medicine, Iowa City, IA 52242, USA; 3 Department of Pathology, The University of Iowa Carver College of Medicine, Iowa City, IA 52242, USA

**Keywords:** p53, acetylation, post-translational modifications, transcription, histone acetyltransferases (HATs), p300/CBP, PCAF, MYST family HATs (Tip60, MOF, MOZ), tumor suppression

## Abstract

Post-translational modifications of p53 are critical in modulating its tumor suppressive functions. Ubiquitylation, for example, plays a major role in dictating p53 stability, subcellular localization and transcriptional *vs.* non-transcriptional activities. Less is known about p53 acetylation. It has been shown to govern p53 transcriptional activity, selection of growth inhibitory *vs.* apoptotic gene targets, and biological outcomes in response to diverse cellular insults. Yet recent *in vivo* evidence from mouse models questions the importance of p53 acetylation (at least at certain sites) as well as canonical p53 functions (cell cycle arrest, senescence and apoptosis) to tumor suppression. This review discusses the cumulative findings regarding p53 acetylation, with a focus on the acetyltransferases that modify p53 and the mechanisms regulating their activity. We also evaluate what is known regarding the influence of other post-translational modifications of p53 on its acetylation, and conclude with the current outlook on how p53 acetylation affects tumor suppression. Due to redundancies in p53 control and growing understanding that individual modifications largely fine-tune p53 activity rather than switch it on or off, many questions still remain about the physiological importance of p53 acetylation to its role in preventing cancer.

## 1. Introduction to p53 and Acetylation

**Figure 1:**
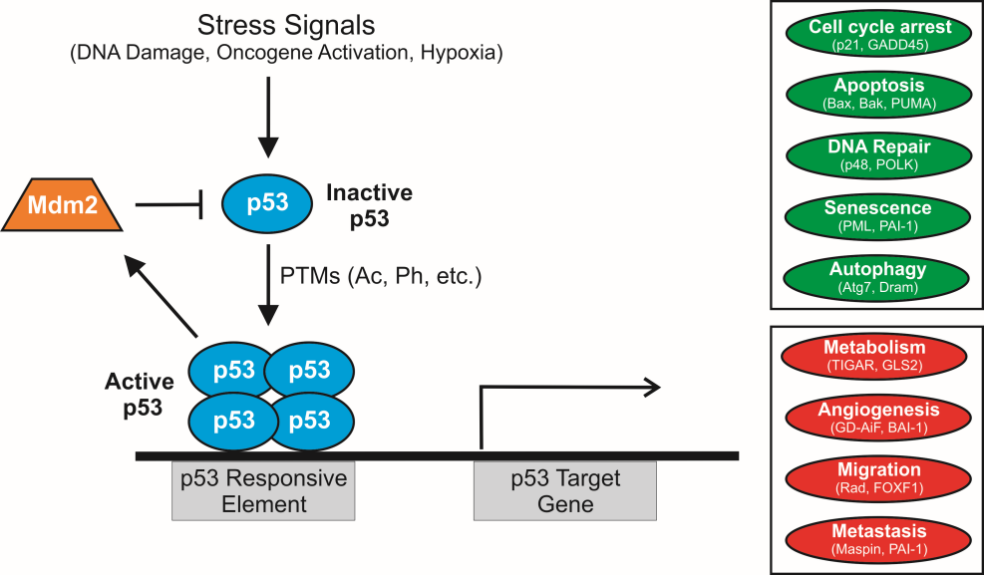
Cellular stress leads to p53 transcriptional activation of downstream targets. Normally, p53 levels are kept low by its major antagonist, Mdm2, an E3 ubiquitin ligase that is itself a transcriptional target of p53. Stress signals, such as DNA damage, oncogene activation and hypoxia, promote p53 stability and activity by inducing posttranslational modifications (PTMs) and tetramerization of p53. p53 functions as a transcription factor that binds to specific p53 response elements upstream of its target genes. p53 affects many important cellular processes linked to tumor suppression, including the induction (green) of senescence, apoptosis, and DNA repair as well as inhibition (red) of metabolism, angiogenesis, and cell migration. These functions are largely mediated through transcriptional regulation of its targets (examples given).

p53 is one of the most studied proteins in science. To date, over 68,000 papers appear in PubMed containing p53 or *TP53* in the title and/or abstract. Originally described as an oncogene (since a mutated, functionally altered form of the protein was first characterized), p53 is now recognized as the most frequently inactivated tumor suppressors in human cancers. It is a transcription factor that controls the expression of genes and miRNAs affecting many important cellular processes including proliferation, DNA repair, programmed cell death (apoptosis), autophagy, metabolism, and cell migration [1,2] (Figure 1). Many of those processes are critical to a variety of human pathologies and conditions extending beyond cancer, including ischemia, neurodegenerative diseases, stem cell renewal, aging, and fertility [1]. Notably, p53 also has non-transcriptional functions, ranging from intrinsic nuclease activity [3] to activation of mitochondrial Bak (Bcl-2 homologous antagonist killer) and caspase-independent apoptosis [4,5,6,7].

As a transcription factor, p53 responds to various genotoxic insults and cellular stresses (e.g., DNA damage or oncogene activation) by inducing or repressing the expression of over a hundred different genes [1,8,9]. p53 transcriptional regulation plays a dominant role in causing the arrest of damaged cells, facilitating their repair and survival, or inducing cell death when DNA is damaged irreparably. p53 can also cause cells to become permanently growth arrested, and there is compelling *in vivo* evidence that these “senescent” cells secrete factors that enhance their clearance by the immune system, leading to tumor regression [10,11]. Through these mechanisms, p53 helps maintain genomic stability within an organism, justifying its long-held nickname “guardian of the genome” [12]. Other p53 gene targets are involved in inhibiting tumor cell angiogenesis, migration, metastasis and other important processes (such as metabolic reprogramming) that normally promote tumor formation and progression [1,9,13,14]. Debate remains about the relative contribution of p53’s transcription-independent activities to tumor suppression [4,5,15]. While those activities are sure to play a role, recent *in vivo* data showing that a transactivation-deficient form of p53 (mutated in both transactivation domains, TAD1 and TAD2) fails to inhibit tumor formation firmly establishes that the transcriptional activity of p53 is essential for tumor suppression [16,17].

**Figure 2:**
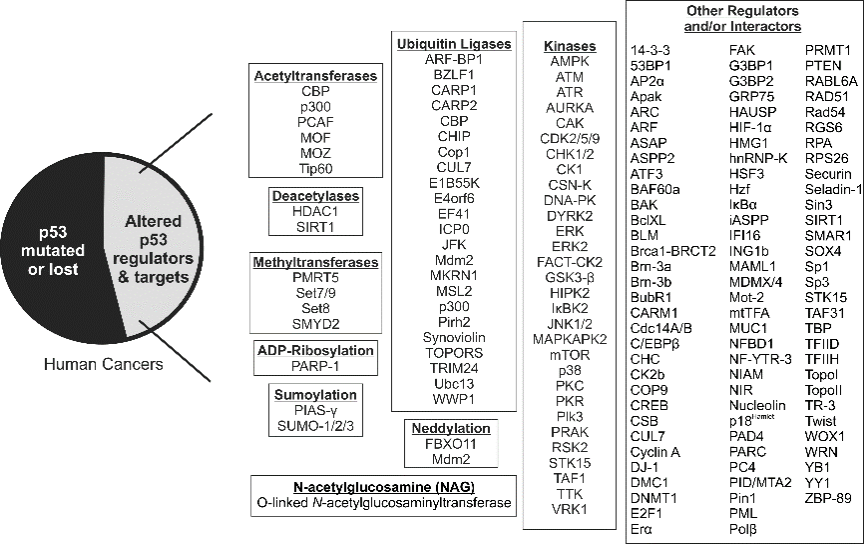
The p53 signaling pathway is altered in the majority of human cancers. The *TP53* gene is deleted or mutated in approximately 55% of human cancers while its signaling is disrupted by alterations to its many regulators (listed to the right) and/or targets (not listed) in the remaining tumors.

Numerous mouse studies have shown that loss of p53 function predisposes cells to permanent damage and neoplastic transformation, greatly increasing the probability of tumor development [18,19,20,21]. In people, the p53 network is inactivated in most, if not all, cancers [2,22,23,24,25,26]. Specifically, the *TP53* gene is deleted or mutated in approximately 55% of sporadic human cancers while p53 signaling is disrupted by alterations to its many regulators and/or targets in the remaining tumors (Figure 2). 

Inherited *TP53* mutation in patients with Li-Fraumeni and Li-Fraumeni-like syndromes also causes a predisposition to early onset cancers including breast carcinomas, brain tumors, leukemias and sarcomas, among others [24,27]. Given its central role in cancer development, great strides have been taken to determine how p53 functions and how it is normally regulated so that treatments can be developed to restore p53 pathway activity in tumors [28,29,30].

The p53 protein is controlled by many different forms of post-translational modifications, including ubiquitylation, phosphorylation, acetylation, sumoylation, methylation, and neddylation [31,32,33,34]. These modifications can dictate the p53 response to diverse cellular signals and help determine its physiological activities. The regulation and effects of p53 ubiquitylation and phosphorylation have been extensively studied and reviewed elsewhere (see above reviews). Comparatively less is known about the other modifications although our understanding of p53 acetylation has grown steadily over the past ten to fifteen years. Acetylation of one or more lysines in a protein can have functional effects by altering its conformation and/or interactions with other proteins. This modification was originally identified on the N-terminal tails of histones and found to neutralize their positive charge, causing decondensation of chromatin and marked changes in gene expression patterns [35,36,37]. Wei Gu and colleagues discovered that p53 can also be acetylated, making it the first non-histone protein shown to undergo that modification [38].

**Figure 3:**
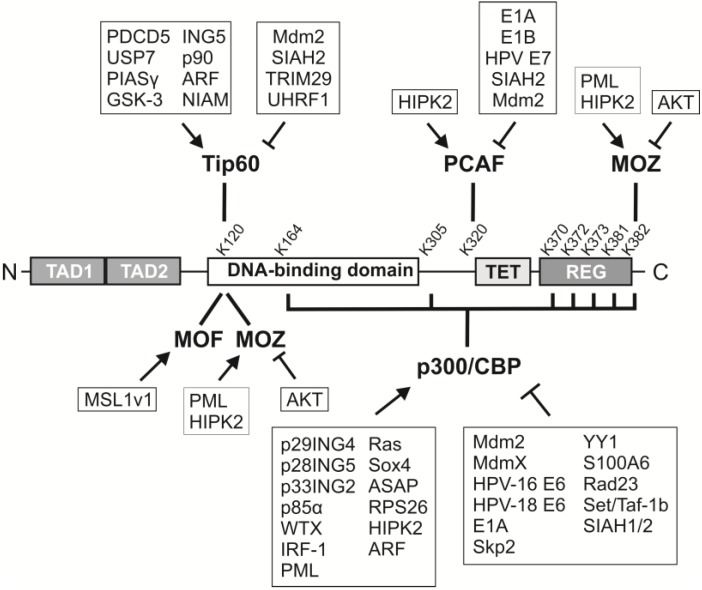
p53 acetyltransferases are targeted by multiple proteins that regulate their function. Schematic of p53 highlighting its acetylation by six different histone acetyltransferases, p300/CBP, PCAF, Tip60, MOF, and MOZ. The ability of these acetyltransferases to regulate p53 is influenced both positively (arrows) and negatively (perpendicular bars) by many types of proteins, including the indicated viral proteins, ubiquitin ligases, kinases, and other cellular factors. TAD, transactivation domain; TET, tetramerization domain, REG, regulatory domain.

**Figure 4:**
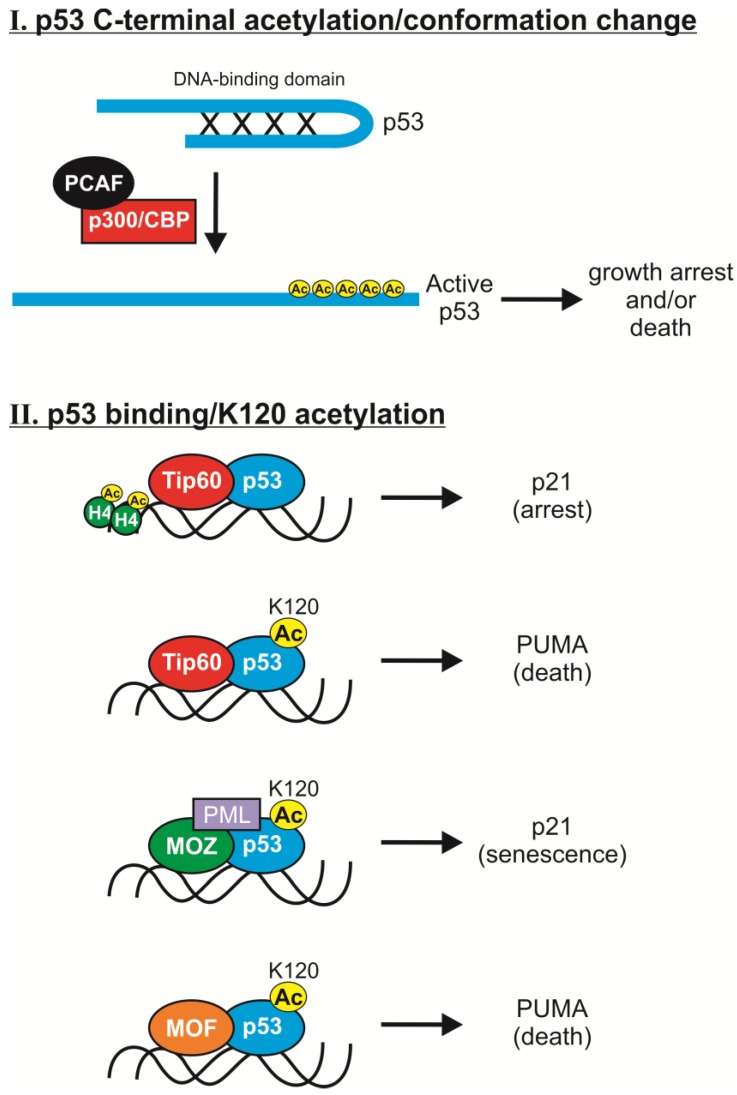
Acetylation-dependent mechanisms of p53 activation. Two primary mechanisms exist by which p53 function is enhanced by its acetyltransferases. (**I**) C-terminal acetylation of p53 by p300/CBP and PCAF promotes an open conformation of p53 by preventing the occlusion of the DNA binding domain by the C-terminal tail. This enhances p53 transcriptional activity, leading to growth arrest and/or apoptosis; (**II**) Interactions of MYST HATs with the central domain of p53 containing K120. Tip60 is an essential component of p53 signaling that activates p53 through direct association on target promoters as well as acetylation of p53 at K120. p21 expression is induced by Tip60-p53 complexes that bind to and promote histone H4 acetylation at the p21 promoter, which leads to a potent growth arrest. K120 acetylation mediated by Tip60 and MOF selectively increases p53 binding to apoptotic gene promoters, thus inducing cell death. Unlike Tip60 and MOF, MOZ-mediated K120 acetylation of p53 specifically induces senescence by promoting transactivation of the p21 promoter. For simplicity, other concomitant p53 acetylation events and association with p300/CBP and/or PCAF are not shown.

Acetylation has many important effects on p53. It increases p53 protein stability, binding to low affinity promoters, association with other proteins, antiviral activities, and is required for its checkpoint responses to DNA damage and activated oncogenes [39,40,41,42]. Six acetyltransferases have been identified that modify p53 at lysines predominantly in the C-terminus or its central DNA binding domain (Figure 3). Acetylation of p53 directly affects its transcriptional activity by opening up its normally closed conformation or by altering its binding to certain response elements in gene targets (Figure 4). In general, these modifications are mediated by two different groupings of acetyltransferases, p300/CBP/PCAF or Tip60/MOF/MOZ. There appears to be significant redundancy in sites of p53 acetylation since loss of one or more sites, including all seven C-terminal lysines in mouse p53, can be largely compensated for by acetylation of remaining lysines [43,44]. However, combined loss of eight major acetylation sites in human p53 (8KR mutant altered at K120, 164, 370, 372, 373, 381, 382 and 386) renders p53 transcriptionally inert and prevents its induction of cell cycle arrest and/or apoptosis [40]. Conversely, in many cell types the inhibition of histone deacetylases (HDACs) that remove acetyl groups from p53 (*i.e.*, HDAC1 and SIRT1) causes increased p53 acetylation and p53-dependent activation of apoptosis and senescence [45]. Together, these findings suggested that acetylation is an essential regulator of the anti-cancer functions of p53.

Several excellent reviews consider various aspects of p53 acetylation [1,31,45,46,47]. This review provides a comprehensive account of the control of p53 acetylation and its effects on p53 transcription and cell fate, with a focus on its acetyltransferases and their regulators, as well as the effect of other post-translational modifications on p53 acetylation. While much more remains to be learned, the role of p53 acetylation in tumor suppression is also considered.

## 2. p53 Acetylation: Sites, Acetyltransferases and Consequences

### 2.1. C-Terminal Acetylation

Initial work revealed that several C-terminal lysines in human p53 (K370, K372, K373, K381, K382) are acetylated by p300 and CBP (CREB-binding protein) [38]. Acetylation of those residues promotes an open conformation of p53 by inhibiting the ability of its C-terminus to bind and occlude the DNA binding domain, thereby enhancing p53 transcriptional activity (see Figure 4) [38,48,49,50,51]. Each of those lysines is evolutionarily conserved across species [52] and their acetylation is induced by various forms of DNA damage, suggesting that p53 acetylation regulates the fate of a cell in response to those signals [39,40,50,51,53,54,55].

p300 and CBP are highly similar proteins with acetyltransferase activity that show approximately 91% homology in their histone acetyltransferase (HAT) domains [56,57,58,59,60]. Consequently, many studies test either p300 or CBP in their experiments while assuming that both contribute to the same functions. Indeed, both p300 and CBP can directly bind to p53 [38,48,49,61,62,63,64,65] and additional studies confirmed they acetylate the C-terminal sites listed above plus two additional lysine residues, K164 and K305 (Figure 3) [40,51,53,54]. Depending on the site(s) modified, acetylation is associated with changes in p53 DNA binding affinity and/or cofactor recruitment to particular p53 response elements. For instance, p53 acetylation at K382 and K320 promotes recruitment of its co-activators, p300/CBP and TRRAP (Transformation/transcription domain-associated protein), to the *p21* (*CIP1/WAF1/CDKN1A*) promoter and increases histone acetylation following DNA damage [66].

Acetylation of p53 by p300 and CBP acetyltransferases is generally considered activating, but that is not always the case. The biological consequences of p53 acetylation by p300 may depend significantly on cellular context, such as cell type and/or transformation status (*i.e.*, primary, immortalized or tumor-derived). As a case in point, p300-mediated acetylation of p53 in human cancer cell lines has been shown to be essential for *p21* promoter transactivation and cell cycle arrest [63,66,67,68]. p21 is a cyclin-dependent kinase inhibitor and important negative regulator of proliferation whose transcriptional upregulation by p53 provokes transient cell cycle arrest or senescence [69,70]. Yet analyses of primary mouse embryo fibroblasts (MEFs) lacking both p300 and CBP revealed those factors are not required for p53-mediated upregulation of *p21* and *Mdm2* following DNA damage, although it is notable that the magnitude of their induction was reduced [71].

Cell type differences also influence the role of p300 in apoptosis. Most work agrees with early findings that p300 is required for p53 acetylation and p53-dependent apoptosis [48]. Numerous cell types were examined in those analyses, including primary MEFs and various human cancer lines. In contrast, loss of p300-mediated p53 acetylation in HCT116 colorectal cells resulted in increased expression of *PUMA* (p53 upregulated modulator of apoptosis) and apoptosis following DNA damage, suggesting p300 expression normally suppresses p53-dependent apoptosis in those cells [67]. Intriguingly, p300 may act similarly in neurons. p53 acetylation at K381 and K382 in neuronal cells specifically inhibits p53 binding to the *PUMA* promoter, preventing PUMA expression and DNA damage-induced cell death [72]. Observations that p53 C-terminal acetylation enhances neuronal survival yet typically causes tumor cell death led the authors to propose that strategies aimed at promoting p53 acetylation (*i.e.*, HDAC inhibition) might be beneficial for preventing neuronal cell degeneration without compromising tumor suppression [72].

Another histone acetyltransferase, PCAF (p300-CBP associated factor), was found to acetylate p53 at lysine 320 (K320) and improve its ability to bind to particular DNA sequences [51,54,73]. Microarray studies showed that K320 acetylation promotes cell survival by selectively inducing expression of anti-apoptotic genes and repressing pro-apoptotic genes [74]. Likewise, results from a mouse knock-in model showed that acetylation of murine p53 at K317 (equivalent to K320 in humans) normally prevents p53-dependent induction of apoptotic genes, *Noxa* (Bcl-2 homology 3-only apoptotic protein) and *Pidd* (p53-Induced protein with a Death Domain), and inhibits apoptosis following DNA damage [75]. Expression of PCAF is also essential for the induction of p21 expression, although this upregulation does not require K320-p53 acetylation [76]. Overall, it has been suggested that PCAF-mediated K320 p53 acetylation suppresses apoptosis and provides the cell with time to repair DNA and continue proliferating if the cellular damage does not reach a critical threshold [74].

### 2.2. K120 Acetylation

While most acetylated lysines in p53 are located in its C-terminus, lysine 120 (K120) resides within the DNA-binding domain and was originally shown by two different groups to play a critical role in promoting p53-mediated apoptosis [77,78]. Acetylation of p53-K120 occurs after exposure to many different types of DNA damage [77,78] as well as expression of the Alternative Reading Frame (ARF) tumor suppressor [79], a major p53 activator that mediates the oncogene checkpoint [23]. Notably, p53-K120 is targeted by missense mutation in many different types of human cancers, implying it plays a vital role in tumor suppression [80,81,82,83,84]. Three histone acetyltransferases in the MYST (Moz, Ybf2/Sas3, Sas2, Tip60) family, Tip60 (HIV-1 tat-interacting protein, 60 kilodalton), MOF (males absent on the first) and MOZ (monocytic leukemia zinc finger) have been shown to acetylate K120 on p53 (Figure 3) [77,78,85]. The enzymes that acetylate the C-terminal lysines in p53, CBP/p300 and PCAF, do not modify K120 [77].

Tip60 is involved in many critical cellular functions, including chromatin remodeling, transcriptional regulation, modification of key signaling proteins, and DNA repair [86]. It can acetylate a variety of cellular proteins, including the ATM/ATR kinases and several different histones (H2AK5, H3K14, H4K5,8,12,16), but has been shown to be a key regulator of the p53 tumor suppressor pathway [46,77,78,86]. Tip60 is required for the p53-dependent induction of *p21* and *PUMA*, and activation of the G1/S DNA damage checkpoint [77,87,88]. Interestingly, Tip60 was first found to activate p53 indirectly through its association with Mdm2 (mouse double minute 2), an E3 ubiquitin ligase and the major antagonist of p53 [88,89,90]. Tip60 prevents the degradation of p53 by Mdm2, not by preventing Mdm2-mediated p53 ubiquitylation but rather by binding and relocalizing Mdm2 into PML (promyelocytic leukemia) bodies where its interaction with the proteasome is impaired [88,89]. Notably, Tip60 can block Mdm2-mediated p53 neddylation [89], a modification known to inhibit its transcriptional activity [91].

Direct control of p53 signaling by Tip60 occurs through both K120 acetylation-dependent and -independent mechanisms. K120 acetylation of p53 by Tip60 is associated with the selective induction of apoptosis rather than cell cycle arrest [77,78]. This site-specific acetylation is required for p53 binding to the *Bax* (Bcl2-associated X protein) and *PUMA* promoters, as well as histone H4 acetylation at the *PUMA* promoter [77,78]. In contrast, acetylated p53-K120 does not accumulate at the *p21* promoter, reflecting the fact that K120 acetylation selectively increases p53 binding to apoptotic gene promoters which are normally low affinity (see Figure 4) [77,78,92,93]. Consistent with those findings, mutation of K120 did not affect the expression of p53 targets with higher affinity response elements (*p21* and *Mdm2*) and had no effect on p53-mediated growth arrest, but it did impair p53-dependent apoptosis [77,78]. These studies suggested that Tip60-mediated p53-K120 acetylation tells a cell with damaged DNA to die rather than arrest and repair its DNA. Importantly, while K120 acetylation is dispensable for *p21* gene regulation, the formation of Tip60-p53 complexes at the *p21* promoter and nearby histone H4 acetylation is required for p53-mediated induction of *p21* expression (Figure 4) [77].Thus, Tip60 is an essential component of p53 signaling that activates p53 through direct association on target promoters as well as acetylation of p53 at K120.

Like Tip60, the MOF HAT acetylates K120 on p53 and specifically transmits an apoptotic response [78,94]. MOF also plays other important roles in gene regulation as the primary acetyltransferase for H4K16 acetylation, which initiates chromatin remodeling and transcriptional regulation [95,96,97,98,99,100]. With regard to p53, however, loss of either Tip60 or MOF decreases the total level of K120 acetylation, establishing both factors as activators of p53 signaling [78]. MOF activity toward p53 is strongly influenced by its binding partners [94]. The interaction of MOF with MSL1v1 (homolog of male specific lethal 1) is significant because it stabilizes MOF-p53 binding and facilitates more efficient p53-K120 acetylation [94]. This in turn results in more effective binding of acetylated p53-K120 to the *Bax* and *PUMA* promoters and increased transcription of those apoptotic genes [78,94]. Conversely, loss of either MOF or its binding partner, MSL1v1, significantly reduces p53-K120 acetylation *in vivo* and decreases expression of *Bax* and *PUMA* [78,94].

The most recent acetyltransferase found to target p53-K120 is MOZ, an additional member of the MYST HAT family. MOZ regulates transcription by promoting histone acetylation as well as through its interaction with proteins such as p53 and p300 [101,102]. Interestingly, unlike Tip60 and MOF, MOZ-mediated K120 acetylation of p53 specifically promotes its anti-proliferative activity. MOZ-p53 complexes bind and promote transactivation of the *p21* promoter in response to DNA damage and oncogene activation, while having no effect on *Bax*, *Mdm2*, and *PUMA* expression [85,102]. In fact, acetylation by MOZ was found to be essential for p53-dependent expression of p21 and senescence in response to oncogenic Ras [85]. This difference in transcriptional regulation by MOZ *vs.* other MYST family HATs could exist because MOZ-p53 complexes are found in PML-NBs (promyelocytic leukemia protein nuclear bodies), suggesting that co-factors and sub-nuclear location of acetylated p53-K120 guides the choice of p53’s transcriptional targets [85]. MOZ also promotes K382 acetylation of p53, unlike Tip60 and MOF, which could likewise influence their differential effects on p53 gene target selection [85,102].

The above analyses of K120 acetylation demonstrate that it significantly influences the specificity of p53 DNA binding, governing its selection of apoptosis *vs.* cell cycle inhibitory genes and thereby dictating cell fate (death *vs.* arrest). Interestingly, the physiological state of the cell may prove important since K120 acetylation under physiological salt concentrations enhances p53 binding to low affinity response elements (*Bax*, *PUMA*) as opposed to high affinity promoters (*p21*) [93].

Considered together, the collective studies of p53 acetylation reveal that stress-induced p53 acetylation within its C-terminus and/or DNA binding domain dictates its binding and regulation of particular gene promoters, leading to distinct molecular and biological consequences. Table 1 summarizes the major effects of different cell stimuli on p53 acetylation status, p53 gene target regulation, and the resulting molecular changes and biological responses. It is clear, however, that these correlations are significantly affected by many variables including (1) type of cellular stress (e.g., DNA damage, hypoxia, oncogene activation, *etc.*); (2) magnitude of the stress and extent of consequent cell damage; (3) type of cell (e.g., epithelial *vs.* neuronal, normal *vs.* transformed); (4) analysis of endogenous or overexpressed p53; and (5) whether or not studies are performed in cultured cells or *in vivo* in mice.

## 3. p53 Deacetylation

Activation of p53 by acetylation suggests that its deacetylation would play an important role in down-regulating p53 transcriptional activity and promoting cell survival following a stress response. In fact, distinct mammalian histone deacetylase (HDAC) complexes containing HDAC1 [103] or SIRT1 [104,105] can deacetylate p53 and strongly repress p53-dependent transcription, thereby reversing p53-mediated cell growth arrest and apoptosis following DNA damage or oxidative stress. p53 is deacetylated at C-terminal lysines K320, K373 and K382 by HDAC1 while SIRT1 acts more specifically on K382. Interestingly, Mdm2 facilitates HDAC1-mediated p53 deacetylation by recruiting HDAC1 to the p53 complex [106]. The loss of p53 acetylation enables the same lysines to be ubiquitylated by Mdm2 [107], resulting in p53 degradation, whereas expression of a dominant-negative HDAC1 increases DNA damage-induced p53 acetylation, stability and transcriptional activity [106]. Other HDACs (2, 3 and 6) may also down-regulate p53 acetylation and function although more evidence is needed to establish those interactions and their biological significance [108,109].

**Table 1:** Molecular and Cellular Consequences Associated with p53 Acetylation ^a^.

Sites of p53 Acetylation	p53 HATs Involved	Concurrent p53 Phosphorylation	Cell Stimulus	Key p53 Gene Targets	Molecular Phenotype	Biological Consequences
None	None	T377, S378	Mitogens	Increased Mdm2,Pirh2	p53 ubiquitylation & degradation	Cell survival & proliferation
C-terminal	P300/CBP, PCAF; binding by Tip60 w/o acetylation	N-terminal (including S15, T18, S20, S33, and S37)	DNA damage, other genotoxic stresses ^b^	Increased p21, GADD45; Decreased Noxa, Pidd	Inhibition of Mdm2-p53 interaction; Inhibition of Cdk activity ^c^	Cell cycle arrest (G1, G2 and/or S phase); DNA repair
K120, K320 and K382	MOZ, PCAF, p300	S15, S20	DNA damage, oncogene activation (e.g., Ras)	Increased p21	Localization of MOZ-p53 complexes in PML-NBs ^d^	Cellular senescence
K120 and C-terminal	Tip60, MOF, p300/CBP, PCAF	S46	DNA damage, other genotoxic stresses ^b^	Increased Bax, Fas, Noxa and PUMA	p53 binding to low affinity, apoptotic gene promoters	Cellular apoptosis

^a^ Incomplete summary highlighting key modifications of human p53 associated with certain molecular and cellular events; ^b^ The magnitude and type of cellular damage may differentially modulate the extent and pattern of p53 acetylation, thereby specifying its biological effects. For instance, more extensive, irreparable stress-induced DNA damage is associated with more complete p53 acetylation and induction of apoptosis; ^c^ p21-mediated inhibition of cyclin-dependent kinases (Cdks), including cyclin D-Cdk4, E-Cdk2 and A-Cdk2 complexes; ^d^ PML-NBs, Promyelocytic leukemia protein-nuclear bodies.

The p53-SIRT1 relationship is somewhat complicated. Many studies support the conclusion that SIRT1 is an important negative regulator of p53. Early work showed SIRT1 co-localizes with p53 in PML nuclear bodies [42,110] where it deacetylates p53 and antagonizes p53-mediated cellular senescence [110]. Others found the DBC-1 (deleted in breast cancer gene 1) tumor suppressor inhibits SIRT1-mediated p53 deacetylation [111,112], while a positive activator of SIRT1 named AROS promotes cell survival in the face of DNA damage by enhancing SIRT1-mediated deacetylation and inactivation of p53 [113]. In addition, tenovin-6, a small molecule inhibitor of SIRT1 promotes p53 hyperacetylation and activation in cancer cells [114]. However, SIRT1 knockout mice display genomic instability and tumorigenesis [115] and SIRT1-null fibroblasts have an extended replicative lifespan, increased proliferation following low level oxidative stress and defective DNA damage response [115,116,117]. This occurs despite a high level of acetylated p53 and may reflect the ability of SIRT1 to promote DNA repair through deacetylation of other targets that help repair damaged DNA including XPA [118], NBS1 [119] and WRN [120]. It has been suggested that deacetylation of p53 by SIRT1 (at K382) may be essential for the induction of cellular senescence in response to minimal DNA damage, helping cells recover from mild genotoxic insults [115]. That notion supports a broader concept that the magnitude and type of cellular damage may differentially modulate the extent and pattern of p53 acetylation, thereby specifying its biological effects (see Table 1).

## 4. Crosstalk between p53 Ubiquitylation and Acetylation

In the absence of cellular stress, p53 is maintained at low levels within cells by the ubiquitin-proteasome degradation pathway. The main E3 ubiquitin ligase and negative regulator of p53 is Mdm2, which is itself a transcriptional target of p53 that functions in a critical negative feedback loop to restrict p53 levels in non-stressed cells (see Figure 1 and Table 1) [121,122,123,124,125]. Mdm2 also represses p53-mediated transcription through association with p53 on the DNA [32]. A homolog of Mdm2, MdmX (or Mdm4), can similarly bind p53 and inhibit p53-dependent transactivation although unlike Mdm2 it lacks E3 ubiquitin ligase activity [126]. Loss of *Mdm2* or *MdmX* in mice causes early embryonic lethality that is fully rescued by *p53* deletion, establishing the importance of the Mdm2/MdmX interplay with p53 in maintaining strict control of p53 levels and activity *in vivo* [126,127,128,129,130]. Indeed, over 15 other ubiquitin ligases besides Mdm2 target p53, including Pirh2 (p53-induced protein with a RING-H2 domain), ARF-BP1 (ARF binding protein 1), Cop1 (constitutive photomorphogenic protein 1) and E4F1 (E4F transcription factor 1) [33,34,131].

While ubiquitylation generally promotes p53 degradation, it also has degradation-independent effects. Specifically, high levels of Mdm2 activity normally lead to polyubiquitylation and degradation of p53, but at low levels Mdm2 induces p53 mono-ubiquitylation, which inhibits DNA binding and promotes the nuclear export of p53 [132,133,134]. One main function of ubiquitylated p53 in the cytoplasm is to interact with Bcl-XL (B-cell lymphoma extra-large), Bak, and Bax in the mitochondria to promote apoptosis through cytochrome c release [7,15,135,136]. Other transcription-independent functions of cytoplasmic p53 include the regulation of autophagy. p53 is able to induce autophagy by promoting activation of AMPK (5' adenosine monophosphate-activated protein kinase) leading to inhibition of the mTOR (mammalian target of rapamycin) pathway [137]. Other studies show that cytoplasmic p53 can inhibit autophagy, suggesting its regulation of autophagy is complex [5,138].

Importantly, ubiquitin ligases negatively regulate the acetylation of p53 through several different mechanisms. First, Mdm2 ubiquitylates the acetyltransferases p300/CBP, PCAF, and Tip60, which targets those proteins for degradation at the proteasome, thereby reducing their expression and ability to acetylate p53 [39,64,90,139,140,141,142]. Other studies have shown that C-terminal lysines of p53 (K370, K372, K373, K381, K382) are competitively targeted for either acetylation or ubiquitylation [38,106,143,144]. Similarly, the E4F1 ubiquitin ligase blocks PCAF acetylation of K320 by promoting the ubiquitylation of the same site [145]. In the same manner, acetylation of those sites blocks their ubiquitylation and enhances p53 stability and activity even in unstressed cells [39,106,107,143]. Lastly, Mdm2 can promote the interaction of p53 with HDAC1, prompting its deacetylation and functional inhibition in a synergistic manner [106].

Additional crosstalk between ubiquitylation and acetylation occurs because some p53 acetyltransferases, p300/CBP and PCAF, are dual-capability enzymes with ubiquitin ligase functions. Specifically, p300/CBP can function as an E4 ubiquitin ligase for p53 and cooperate with Mdm2 to promote p53 polyubiquitylation [146,147]. Whether or not p300/CBP functions as an acetyltransferase *vs.* ubiquitin ligase of p53 may depend on its localization within the cell since p300/CBP acetylates nuclear p53 but promotes p53 degradation in the cytoplasm via ubiquitylation [146]. Unlike p300/CBP, PCAF functions as an E3 ubiquitin ligase for Mdm2, not p53 [148]. PCAF expression is essential for reducing Mdm2 levels and preventing p53 destabilization in response to UV damage. Importantly, both the HAT and E3 ligase activities of PCAF were required to promote p53-dependent transcriptional activation, which in this study was linked with specific regulation of *NOXA* and apoptosis [148]. Together, these studies indicate that a finely orchestrated balance exists between ubiquitylation and acetylation of p53.

## 5. Effects of p53 Phosphorylation on Acetylation

p53 phosphorylation in response to DNA damage and other cellular stresses has been shown to control its association with regulators, subcellular localization, stability and transcriptional activation or repression of target genes that elicit its biological effects [31,32]. Importantly, the collective phosphorylation changes at multiple residues in conjunction with other post-translational modifications (such as ubiquitylation and acetylation) appear critical in determining p53 function and cell fate whereas individual modifications have less pronounced effects, if any, when modeled *in vivo* [31,32,45].

Many different kinases target specific serine and threonine residues within p53 for phosphorylation. ATM (ataxia telangiectasia mutated), ATR (ataxia telangiectasia and Rad3 related), and DNA-PK (DNA-dependent protein kinase) are each activated by various forms of DNA damage and directly phosphorylate S15 of p53 [149,150,151,152,153,154,155]. S37 is targeted by ATR, DNA-PK, and Chk2 (checkpoint kinase 2) [51,151,152,154], whereas S20 of p53 is a target of both the Chk1 and Chk2 kinases [156,157,158,159]. Chk1 and Chk2 can also phosphorylate additional p53 residues including T387 by Chk1, T18 and S366 by Chk2, and 4 residues (S313, S314, T377, S378) in the C-terminus by both kinases [31]. Other kinases traditionally recognized for their roles in cell cycle progression or gene transcription, such as CAK (cyclin-dependent kinase activating kinase) or HIPK2 (homeodomain interacting protein kinase 2), can phosphorylate p53 at S33 or S46, respectively [160,161,162]. This incomplete listing of kinases that act on p53 in response to different stimuli illustrates the complexity of its control by phosphorylation.

Changes in p53 phosphorylation either facilitate or inhibit the binding of specific interactors to p53, which can then block or promote acetylation. For example, N-terminal phosphorylation of p53 at S15, T18, S20 and/or S37 blocks Mdm2 association, with multisite phosphorylation more effective than isolated phosphorylation events [141,152,156,163,164]. The consequent stabilization of p53 accompanied by reduced modification of lysines with competing ubiquitin fosters increased acetylation. Additionally, N-terminal p53 phosphorylation enhances binding to its acetyltransferases. Phosphorylation of S15, T18, S20 and S37, and/or multisite phosphorylation of p53 promotes the recruitment of p300/CBP [141,163,164,165,166,167,168,169,170], while PCAF-p53 binding is enhanced by S33 and S37 phosphorylation [51]. These findings are consistent with evidence that p53 phosphorylation at sites within its N-terminus is required for K320 and K382 acetylation [161,171,172]. Similarly, phosphorylation of p53 at S15 and S20 promotes its association with MOZ [102], implying it would heighten p53 K120 acetylation.

Several studies have explored the transcriptional and biological consequences of coordinated phosphorylation and acetylation events on p53. Puca *et al.* demonstrated that HIPK2-mediated S46 phosphorylation of p53 is required for p53-K382 acetylation, efficient p300-p53 co-recruitment onto apoptotic promoters following DNA damage, and apoptosis [172]. That agrees with an earlier observation that HIPK2 down-regulation impairs p53-dependent transcription and UV-induced apoptosis [161]. Interestingly, little evidence for crosstalk between p53 phosphorylation and acetylation was seen when endogenous mouse *p53* was mutated at S18 (equivalent to S15 in human) in embryonic stem (ES) cells [173]. Loss of phosphorylation at that residue did impair DNA damage-induced p53 stabilization and p21 expression, as predicted due to increased Mdm2 binding, but it unexpectedly had no effect on C-terminal acetylation at K317 and K379 (corresponding to human K320 and K382). Cell type and transformation status (primary ES cells *vs.* human cancer cell lines) and other variables, such as analysis of exogenous *vs.* endogenously expressed mutant p53, may explain the absence of phosphorylation-acetylation crosstalk in this study *vs.* earlier investigations. Alternatively, given the redundancies in p53 regulation [31,32], phosphorylation of other N-terminal sites may simply compensate for the loss of S18 phosphorylation and support C-terminal acetylation.

While most studies have assessed effects of N-terminal p53 phosphorylation on C-terminal acetylation, Ou *et al.* identified multiple phosphorylated sites in the C-terminus of p53 that negatively affect its nearby acetylation [174]. They found that mutation of several of those sites, T377 and S378, to alanine resulted in greater acetylation of K373 and K382 while having no effect on K120 acetylation. The increased acetylation at K373 and K382 correlated with increased association of the phosphorylation-deficient p53 mutant (T377A/S378A) with p300. Those results suggest that a unique interplay exists between C-terminal phosphorylation and acetylation events on p53 that contrasts with the enhancement of p53 acetylation by N-terminal phosphorylation. Table 1 highlights the different effects of certain p53 phosphorylations on its acetylation, transcriptional activation and cellular consequences.

## 6. Acetylation *vs.* Other Post-Translational Modifications

The impact of other p53 post-translational modifications, such as methylation, sumoylation, and neddylation, on its acetylation and functions is generally not well defined. Methylation has been shown to counteract, cooperate with or not affect p53 acetylation and activation depending on the site of modification and the study. p53 is methylated at K370 by Smyd2 (Set/MYND Domain-2), K372 by Set 7/9 (Su(var)3-9 and “Enhancer of zeste” protein 7/9), and K382 by Set8 (Su(var)3-9 and “Enhancer of zeste” protein 8) [175,176,177,178,179]. Methylation of K370 and K382 inhibits p53 DNA binding and transcriptional activity, and K382 methylation was shown to impair K382 acetylation [176,178]. By comparison, early studies of K372 monomethylation suggested it was essential for efficient p53 transactivation and induction of cell cycle arrest and death [179]. This correlated with findings that K372 methylation promotes C-terminal acetylation of K373 and K382 in response to various stresses [177] while impairing the repressive K370 methylation by Smyd2 [176]. Analyses of Set7/9-null mice initially supported those cell line-based studies. Specifically, Kurash *et al.* showed that loss of the methyltransferase abrogated p53 acetylation at multiple sites, impaired p53-dependent transcriptional activation (of *p21* and *PUMA*), and greatly reduced induction of cell cycle arrest and apoptosis in response to DNA damage and oncogenic stress [175]. These studies indicated a prerequisite role of K372 methylation in p53 acetylation and activation that would be important for tumor suppression.

The above conclusion has not been supported by recent work. Two independent groups have since developed additional *Set7/9* knockout mice and demonstrated no role for that methyltransferase in p53 acetylation (at mouse K379, equivalent to human K382), transcriptional activation or tumor suppression [180,181]. Those findings suggested that Set7/9 is dispensable for p53 function. The reasons for such contradictory results from the different mouse studies are not clear. One suggestion by Campaner *et al.* was that compensatory mechanisms, possibly other p53 methyltransferases that can modify mouse K369, exist in their Set7/9 mutant mice but are somehow absent in the knockout strain developed by Kurash *et al.* [180]. In that regard, antibody limitations have prevented a conclusive demonstration by any group that K369 methylation of p53 is truly lost in Set7/9-deficient mice [180,181]. Given these uncertainties, the physiological relevance of p53 C-terminal methylation to p53 acetylation and activity seems unresolved.

We currently know very little about the effects of p53 sumoylation on its acetylation. In fact, the functional consequences of sumoylation of p53 activity in general remain unclear. Some early reports indicated that sumoylation promotes p53 recruitment into PML nuclear bodies and transcriptional activation [182,183,184]. However, a later study saw no effect of K386 SUMO-1 modification on p53-regulated transcription or subnuclear localization [185] and others found that p53 sumoylation by the SUMO E3 ligase PIASy (protein inhibitor of activated STAT protein gamma) causes its nuclear export [186,187]. In the Naidu *et al.* study, sumoylation of both p53 and Tip60 by PIASy was shown to augment p53 K120 acetylation, promoting p53 cytoplasmic accumulation and induction of PUMA-independent autophagic cell death [187]. It was proposed that coordinated K386 sumoylation and K120 acetylation of p53 acts as a “binary death signal” in which the dually modified p53 is directed to the nuclear pore complex and K120 acetylation specifically helps target p53 to mitochondria. Such a model actually suggests a transactivation-independent role for K120 acetylation. Regardless of the underlying mechanisms, the recent findings support a view that sumoylation generally correlates with impaired transcriptional activity [188]. In agreement, Wu *et al.* found that K386 sumoylated p53 binds p300 but fails to undergo p300-mediated acetylation at K373 and K382, which is associated with an inability to bind DNA and transcribe its gene targets [189]. The same study showed that a sumoylation-deficient p53 mutant increased the transactivation of endogenous *p21* compared to wild-type p53. Interestingly, acetylation restored binding of sumoylated p53 to DNA, suggesting that acetylation can antagonize the inhibitory effect of sumoylation on p53 DNA binding.

Neddylation of p53 has not been thoroughly studied and is poorly understood. It can occur on K370, K372, and K373 by Mdm2 and on K320 and K321 by FBXO11 (F-box protein 11), and in each case it is associated with inhibition of p53-mediated transcription [91,190]. The effect of p53 neddylation on its acetylation remains to be determined but it is predicted to interfere since both modifications occur on the same lysine residues. It is anticipated that Mdm2 and FBX011 will compete with the acetyltransferases for modification of p53 at those sites in response to different cellular signals.

## 7. Regulators of Acetyltransferases

### 7.1. p300/CBP

There are several positive regulators of p300/CBP that induce its acetylation of p53 at various lysines (Figure 3). This includes the actions of three different members of the ING (inhibitor of growth proteins) family. Both p29ING4 and p28ING5 can form complexes with p300 and p53 and induce K382 acetylation, resulting in increased p21 expression as well as induction of apoptosis [191]. p33ING2 also enhances the p300-p53 interaction and increases p300-mediated K382 acetylation of p53, although this is associated with p53-dependent cellular senescence [192,193]. Another study showed that p85α (regulatory subunit of phosphoinositide 3-kinase, p85alpha), a novel interactor of p300, is essential for p300-mediated acetylation of K370 in mouse p53 (equivalent to K373 in humans) and UV-induced apoptosis [194]. In response to UV exposure, p85α promotes p300-p53 binding and recruits those complexes to essential promoters [194]. Two other regulators of p300/CBP, WTX (Wilms’ tumor gene on the X chromosome) and IRF-1 (interferon regulatory factor 1), promote p53 acetylation at K373 and K382 [195,196]. IRF-1 is required for the increase of p300-mediated acetylation of p53 bound to the *p21* promoter, leading to p21 activation [195]. WTX promotes p53 acetylation by preventing p300/CBP destabilization and enhancing CBP-p53 binding [196].

Various factors involved in sensing cellular stresses and mediating p53-dependent checkpoints have been shown to promote p300/CBP acetylation of p53. For instance, the PML tumor suppressor is upregulated in response to oncogenic Ras and is required for triggering a p53-dependent premature senescence that protects cells against Ras-mediated transformation [42]. As part of that process, PML forms a stable complex with p53-CBP within PML nuclear bodies and promotes p53 acetylation at K382. Those events, as well as Ras-induced senescence, are lost in PML-null fibroblasts. A relatively new DNA damage response sensor, Sox4 (sex-determining region Y-related high mobility group box 4), has been similarly found to facilitate p300-p53 association and enhance p53 acetylation [197]. Specifically, RNAi analyses showed that Sox4 is required for maximal p53 acetylation (at K373 and K382), transcriptional activation and induction of cell cycle arrest and apoptosis following DNA damage. Two other newly recognized factors shown to regulate p53 K382 acetylation as part of the DNA damage response include ASAP (aster-associated protein) and RPS26 (ribosomal protein S26) [198,199]. ASAP is a centrosome- and spindle-associated protein that is transiently induced by DNA damage, interacts with p53 and p300, and stabilizes p53 by promoting its acetylation via p300 and blocking it ubiquitylation by Mdm2 [198]. RPS26, like many other ribosomal proteins, can interact with Mdm2 and thereby affect p53 stability [199]. However, RPS26 also forms a complex with p53 and p300 (independent of Mdm2) and its knockdown was found to impair p53 acetylation and recruitment to its target gene promoters in response to DNA damage, which correlated with failed cell cycle arrest.

The HIPK2 kinase promotes p300-mediated acetylation and activation of p53 through at least two different mechanisms. First, HIPK2 can directly phosphorylate p300, which was found to generally stimulate its acetyltransferase activity [200]. While that study did not evaluate effects of HIPK2 on p53 acetylation, a later report demonstrated that HIPK2 induces p53 K382 acetylation via p300, which is required for a maximal apoptotic response [172]. To achieve full HIPK2-dependent activation of pro-apoptotic gene transcription, p53 had to be at least dually modified by S46 phosphorylation (which can be mediated by HIPK2) and K382 acetylation. Silencing of HIPK2 significantly reduced K382-p53 acetylation, impaired recruitment of p53 and p300 to apoptotic gene promoters, and decreased p53 apoptotic transcription. Interestingly, p300 expression rescued the phenotype of HIPK2 knockdown cells and restored K382 acetylation, S46 phosphorylation (presumably by other kinases), and p53-dependent apoptotic gene expression and cell death in response to adriamycin. This suggests that while HIPK2 can enhance p300 regulation of p53, it is not required for its activity.

Many different mechanisms exist to decrease p53 activation by CBP/p300-mediated acetylation, with Mdm2 and its homolog MdmX playing a prominent role. Mdm2 directly binds p300 and CBP and forms a multimeric complex with p53-p300/CBP [39,64,139,140,141]. In so doing, Mdm2 blocks p53 acetylation by p300/CBP, impairing sequence specific DNA binding and transcriptional activation by p53 [39,139,140]. Conversely, the ARF tumor suppressor binds Mdm2 and counteracts its inhibition of p53 acetylation by allowing maintenance of p300-p53 association [39]. While Mdm2 and MdmX can bind to each other, MdmX has been shown to inhibit p300/CBP-mediated p53 acetylation by directly associating with p53 independent of Mdm2 [201]. Notably, two ubiquitin ligases, SIAH1 (seven in absentia homolog 1) and SIAH2 (seven in absentia homolog 2), also target p300/CBP for ubiquitylation and degradation [202].

Viral proteins can be important inhibitors of p300/CBP. Human papilloma viruses, HPV-16 and HPV-18, express E6 proteins that interfere with p53 activity via two mechanisms. The best recognized activity of E6 is its promotion of p53 ubiquitylation and subsequent degradation [203]; however, it can also bind to p300/CBP and interfere with its association with p53 [204,205]. The adenoviral oncoprotein, E1A, likewise prevents p53 acetylation and transcriptional activation by p300/CBP [65,206]. It can do so by sequestering endogenous CBP from p53 [65], as well as displacing PCAF and other co-activators from p300/CBP (thereby directly repressing its acetyltransferase activity toward p53 as well as its other substrates) [206]. Interestingly, E6 proteins from low risk HPV-6 and HPV-11, which are not associated with cancer in people, are not able to bind CBP [205].

Other negative regulators of p300/CBP-mediated p53 acetylation include Skp2 (S-phase kinase associated protein 2), YY1 (yin yang 1), and S100A6 [207,208,209]. Skp2 is an F box protein within the SCF (Skp, Cullin, F**-**box containing) ubiquitin ligase complex that targets various tumor suppressors (including p21) for degradation while conversely promoting the activity of oncoproteins (such as c-Myc), consistent with its overexpression in various human cancers [210]. Skp2 binds the cysteine-histidine-rich (CH1 and CH3) domains of p300 that normally mediate p53 association, thereby blocking the p300-p53 interaction, suppressing p53 K382 acetylation and reducing the transactivation of both apoptotic and cell cycle arrest targets in various cancer cells following DNA damage [207]. YY1 is a multifunctional transcription factor that significantly inhibits the transcriptional activity and checkpoint responses of p53 to DNA damage [208]. It binds p53 and competes for its association with p300, thereby impairing p53 acetylation. YY1 also enhances Mdm2-mediated ubiquitylation of p53. S100A6, a cytosolic calcium binding protein, normally associates with non-acetylated cytosolic p53 and prevents its acetylation by competing with p300 for binding to p53 [209]. The common theme for these factors is that they disrupt p300/CBP-p53 association via interaction with one of the partners in the complex.

Additional negative regulators of p300 include Rad23 (radiation sensitive protein 23) protein A, also called hHR23A, a human homolog of the yeast nucleotide excision repair protein Rad23 [211]. While the exact effects of hHR23A on p53 acetylation remain to be determined, hHR23A was found to bind p300 and its overexpression inhibited p53 transcriptional activity whereas concomitant expression of p300 or CBP overcame that inhibition. Set/Taf-1b (transcription initiation factor TFIID subunit 1b) is another repressor of p53 transcriptional activity that prevents p300- and PCAF-mediated p53 acetylation, resulting in increased cell proliferation under basal growth conditions [212]. Set/Taf-1b also impairs the induction of cell cycle arrest and apoptosis by p53 after cell stress. Studies in *Drosophila* likewise show that Set/Taf-1b inhibits p53 acetylation and apoptosis *in vivo* [212]. In sum, the complex regulation of p300/CBP stability and acetyltransferase activity by numerous cellular and viral proteins underscore its importance in controlling p53 activity and transcriptional activation.

### 7.2. PCAF

Like p300/CBP, PCAF activity towards p53 is regulated by both viral and mammalian proteins (Figure 3). This includes negative regulation by adenoviral E1A and E1B proteins, which prevent PCAF-mediated acetylation and activation of p53 [206,213,214], thereby removing one of the strongest cellular barriers to viral-induced transformation. E1A can compete with PCAF for p300/CBP binding, perhaps preventing PCAF recruitment to certain promoters, and thus antagonize PCAF-mediated cell cycle arrest [73]. E1B interrupts the interaction between PCAF and p53 and prevents p53 from binding to specific DNA sequences that would normally be promoted by its acetylation [213]. Both high and low risk HPV E7 proteins can bind PCAF and potentially inhibit its acetyltransferase activity [214].

Most of the cellular factors that act on PCAF also directly regulate p53. For example, SIAH2 and Mdm2 are able to bind and ubiquitylate PCAF, promoting its degradation at the proteasome [142,202,215]. These events directly reduce PCAF-mediated acetylation of p53 and interrupt its transcriptional activation [142,202]. HIPK2 is a positive regulator of PCAF that interacts with both p53 and PCAF [161]. As part of checkpoint responses to cisplatin, HIPK2 induces PCAF-mediated acetylation of p53 at K320, which leads to p53-dependent transactivation of *p21* but not other targets (e.g., *p53AIP1*) [161,216]. Increased levels of acetylated K320-p53 and H4 acetylation were found at the *p21* promoter when HIPK2 was present [216]. Notably, increased K320 acetylation of p53 was only associated with the growth inhibitory dose of cisplatin, not the dose that induces apoptosis. This supports the idea that the type and amount of stress can engage certain acetylation events and determine the affinity of p53 for specific transcriptional targets (see Table 1).

### 7.3. Tip60

The Tip60-p53 node is controlled by various mechanisms that likewise involve post-translational modification of Tip60 or association with co-factors that influence Tip60-p53 complex formation (Figure 3). Which mechanism(s) dominates is expected to reflect the expression levels of the different Tip60 regulators and the particular stress conditions occurring within the cells (DNA damage, oncogene activation, *etc.*).

Ubiquitylation of Tip60 plays an important role in its regulation. Various E3 ubiquitin ligases, including Mdm2, SIAH2 and TRIM29 (tripartite motif-containing protein 29) mediate Tip60 polyubiquitylation and promote its proteasomal degradation [90,202,217]. The resulting decrease in Tip60 levels would clearly impair its association with p53 and ability to promote K120 acetylation. Interestingly, UHRF1 (ubiquitin-like with PHD and RING finger domains 1) was recently identified as a new critical negative regulator of Tip60, which acts at least in part by inducing degradation-independent ubiquitylation of Tip60 [218]. UHRF1 expression blocked K120 acetylation of p53 by Tip60 whereas its depletion significantly increased K120 acetylation and p53-dependent apoptosis. UHRF1 binds directly to Tip60 through its SRA (Set and RING-associated) and RING (really interesting new gene) domains, and in so doing markedly inhibits Tip60-p53 complex formation. As a consequence, UHRF1 reduced the expression of p53 target genes, such as *p21*, which depend on p53-Tip60 binding at the promoter rather than p53 K120 acetylation [77]. Thus, UHRF1 inhibits Tip60 activity toward p53 through both K120 acetylation-dependent and -independent mechanisms.

The inhibition of Tip60 by various ubiquitin ligases is countered by the opposing effects of several deubiquitinases, PDCD5 (programmed cell death 5) and USP7 (ubiquitin-specific-protease 7). Both factors enhance Tip60 stability by decreasing its ubiquitylation and preventing its proteasomal degradation [219,220]. PDCD5 expression was found to induce Tip60-mediated K120 p53 acetylation as well as acetylation of H2A, H3, and H4 [219]. After DNA damage, PDCD5 is required for p53-mediated induction of *Bax,* thus promoting Tip60-dependent apoptosis [219]. USP7 also increases Tip60-dependent p53-K120 acetylation, which is required for its induction of the pro-apoptotic gene, *PUMA* [220]. Both deubiquitinases therefore promote apoptosis by positively regulating Tip60 stability and ability to acetylate its targets, including p53.

Other post-translational modifications of Tip60 are associated with its activation and increased p53-K120 acetylation. For example, Tip60 sumoylation by PIASy enhances its acetylation of p53-K120; however, the coincident sumoylation of p53 by PIASy uniquely targets the K120-acetylated form to the cytoplasm where it acts in a transactivation-independent manner to induce autophagy in response to DNA damage [187]. Tip60 phosphorylation also promotes its acetyltransferase activity. GSK-3 kinase (glycogen synthase kinase 3) phosphorylates Tip60 at S86 in response to DNA damage, thereby inducing p53-K120 acetylation, H4 acetylation at the *PUMA* promoter, increased *PUMA* expression, and apoptosis [221]. Interestingly, GSK-3 phosphorylation of S86 on Tip60 requires a priming phosphorylation at S90 by an unknown kinase [221].

Co-regulators of Tip60 have been identified that exert their effects via protein-protein interactions that promote p53-Tip60 complex formation and K120 acetylation. ING5, a member of the inhibitor of growth (ING) family, is a good example. It is upregulated in response to DNA damage, forms a complex with p53 and Tip60, and selectively promotes the acetylation of p53 at K120 by Tip60 (but not by its relative, MOF) [92]. This causes increased expression of p53 apoptotic target genes, such as *BAX*, and ultimately cell death. The effects of ING5 are abolished by K120R mutation, showing they are dependent on K120 acetylation. Another regulator of p53 apoptotic genes is p90 (coiled-coil domain containing 8), which can bind Tip60 directly and promote Tip60-mediated K120 acetylation on p53 [222]. p90 selectively induced the expression of *PUMA*, not *p21*, and increased p53-dependent apoptosis.

Several p53 activators have been shown to bind and regulate Tip60 although their effects on Tip60-mediated acetylation of p53 are unclear. For example, RGS6 (regulator of G protein signaling 6) is a newly identified activator of p53-dependent apoptosis in response to doxorubicin (adriamycin) [223]. Separate work showed it can bind Tip60 and block Ras-induced transformation by facilitating Tip60 acetylation and subsequent degradation of Dnmt1 (DNA methyltransferase 1), ultimately promoting apoptosis [224]. Based on those observations, it is plausible that RGS6 could promote Tip60-mediated K120 acetylation of p53, although that remains to be tested. Another p53 regulator whose role in Tip60-dependent p53 acetylation is undefined is the ARF tumor suppressor. ARF is an essential activator of p53 in response to oncogene activation that has been shown to interact directly with Tip60 and increase its stability [225,226]. The ARF-Tip60 interaction was actually found to be important for p53-independent ARF signaling following DNA damage [226], but others had reported that ARF overexpression can promote p53 K120 acetylation [79]. The assumption from those independent findings is that p53-K120 acetylation by ARF would be mediated by Tip60, however that has not been shown. MOF and MOZ also promote K120 acetylation of p53 [77,78,85], and MOZ has been specifically linked with Ras-induced K120 acetylation and senescence [85]. Given ARF’s key role in Ras signaling and senescence, more studies are needed to determine the significance of ARF-mediated Tip60 regulation (or possibly MOZ) in controlling p53 acetylation and activation.

A more recently identified positive regulator of Tip60 is NIAM (Nuclear Interactor of ARF and Mdm2, gene name *Tbrg1*), a novel chromatin bound protein that inhibits proliferation and promotes p53 signaling [227,228]. New evidence reveals that NIAM activates p53 through multiple mechanisms involving Mdm2 inhibition and Tip60 association [228]. NIAM expression was sufficient to induce K120 acetylation of p53 and Tip60 depletion impaired NIAM-mediated p53 activation. However, the use of NIAM mutants showed that its association with Tip60, not its induction of p53-K120 acetylation, was essential for maximal p53-dependent activation of the *p21* promoter. These data supported a model wherein NIAM enhances the binding ofTip60 to non-K120-acetylated p53 at the *p21* promoter, and possibly increases nearby histone H4 acetylation (see Figure 4) [77]. Indeed, Tip60 association with p53 in the absence of K120 acetylation induces p21 expression and cell cycle arrest (Table 1), consistent with the G1 arrest promoted by NIAM.

### 7.4. MOF and MOZ

The regulation of p53 acetylation by MOF and MOZ has not been as well studied as that for p300/CBP, PCAF and Tip60. MOF is positively regulated toward p53 thru its interaction with MSL1v1, which stabilizes the MOF-p53 complex and facilitates p53-K120 acetylation (Figure 3) [78,94]. By comparison, MOZ colocalizes with p53 and PML in PML-NBs in response to cellular stress, which enhances its acetylation of p53 at not only K120 but also K382 [85]. Conversely, an Akt (Ak mouse strain thymoma protein) / protein kinase B phosphorylation site exists within the PML binding domain of MOZ and phosphorylation at that site (T369) by Akt inhibits MOZ association with PML and p53 acetylation. MOZ is also a co-activator for AML1 (acute myeloid leukemia 1 protein) and forms complexes containing AML1, PML and other HATs, such as p300/CBP, to activate transcription [229]. Therefore, it may be significant that HIPK2 can also interact with those complexes and phosphorylate MOZ [200], as it suggests another possible mechanism by which MOZ-mediated acetylation of p53 might be controlled. Little else is known regarding MOF or MOZ regulation as it relates to their acetylation of p53, indicating that more studies are needed to determine the identity and function of regulators controlling these two p53 acetyltransferases.

## 8. Role of p53 Acetyltransferases in Development and Cancer

Since acetylation appears to be required for p53’s anti-proliferative and pro-apoptotic functions, it is not surprising that many of its acetyltransferases are lost or mutated in cancer. For example, p300/CBP is important for preventing the development of hematologic malignancies [230,231,232] and is mutated in solid tumors, such as gastrointestinal tumors [232,233,234,235]. Microarray analyses revealed reduced PCAF mRNA levels in several types of cancer, including colon, lung, and bladder [236], which was supported by immunohistochemical data showing decreased PCAF protein expression in human intestinal type gastric cancer and colorectal tumors [236,237]. Tip60 is a haploinsufficient tumor suppressor in mice and loss of one allele is observed in low-grade lymphomas and breast cancers in people [238]. *Tip60* expression is also significantly lower in human gastric, colon and lung cancers as well as several other malignancies, including leukemia and melanoma [239,240,241,242,243]. In melanoma patients, reduced Tip60 protein expression is associated with poor survival, perhaps reflecting the ability of Tip60 to inhibit melanoma cell migration and increase chemosensitivity [243]. In hematopoietic cells, Tip60 negatively regulates transcriptional activation by the oncogene c-Myb (myeloblastosis protein) by facilitating its association with the histone deacetylases HDAC1 and HDAC2 [242]. This observation suggests that Tip60 expression is important for inhibiting c-Myb mediated leukemogenesis. Interestingly, MOZ, CBP and p300 are frequently altered by chromosomal translocations in human leukemias, yielding chimeric fusion proteins that promote leukemogenesis through inhibition of hematopoietic cell differentiation and increased progenitor cell self-renewal [101,234,244,245,246,247]. These findings support tumor suppressor roles for each of these acetyltransferases in cancer.

MOF status is not as well studied as the other p53 acetyltransferases in human tumors and its role in suppressing *vs.* promoting oncogenesis is more ambiguous. On the one hand, depletion of MOF in various tumor cell lines causes a variety of phenotypes (genomic instability, reduced proliferation and defective ATM-p53 mediated checkpoint responses) consistent with a tumor suppressive role [248]. On the other hand, MOF expression was found to be elevated in a significant fraction of human tumor cell lines and primary tumors, and its overexpression caused increased proliferation, oncogenic transformation and enhanced tumor growth in mouse xenografts [249]. While MOF’s acetyltransferase activity can increase p53 transcriptional activity and apoptosis [78], it has other biologically relevant substrates whose altered acetylation by aberrant MOF expression could explain these opposing effects. MOF is the major acetyltransferase that modifies histone H4 at K16 and as such it is a critical regulator of chromatin structure and transcription [97,99,100,248,249,250]. It was therefore suggested that MOF’s oncogenic activities reflect its regulation of H4K16 acetylation [249].

We have focused on p53 regulation by these acetyltransferases, yet it is clear that all of these enzymes target other substrates in addition to p53, most notably histones. Besides histone H4K16 regulation by MOF, for example, Tip60 modification of histones H2AX and H4 is essential for the decondensation of chromatin required for sensing and repairing DNA damage [251]. CBP and p300 can acetylate all four histones [232] although biochemical analyses of H3 and H4 acetylation reveal different specificities of each enzyme for certain lysines on those histones [252]. Moreover, CBP and p300 acetylate numerous non-histone proteins besides p53, including p73, Rb, E2F, myb, myoD, GATA1 among others, not to mention their role as co-activators within many different transcriptional complexes [232]. Therefore, when we consider the role of p53 acetyltransferases in cancer it must take into account their far-reaching influence on other vital cellular processes. The same must be done when considering their importance in development where each acetyltransferase, with the exception of PCAF, has been shown to be essential. Early embryonic lethality in mice results from the genetic deletion of *p300*, *CBP*, *Tip60*, *MOF* or *MOZ* [249,253,254,255]. Importantly, concomitant deletion of *p53* failed to rescue the embryonic death caused by *MOF* ablation, establishing the p53-independence of that phenotype [249].

The lethality associated with loss of the p53 acetyltransferases is notably distinct from the effect of *p53* loss *in vivo*, which is generally well tolerated outside of the predisposition to cancer. While a small fraction of p53-deficient embryos develop exencephaly due to neural tube defects and excessive neural tissue growth [256], and depending on the strain some p53-null mice have vision defects [257,258,259], *p53* knockout mice are viable and generally develop normally [18,19]. That is consistent with the fact that the p53 network is normally kept “off” in most cells and is only activated in response to particular cell stresses or signals [1,2,22]. By comparison, unrestrained expression and pro-apoptotic activity of p53 are more detrimental to embryogenesis, as shown by the early embryonic lethality of mice lacking *Mdm2* or *MdmX* and rescue of that phenotype by *p53* deletion [21,127,128,129,130,260]. Together, these findings illustrate the unique effects of p53 and its acetyltransferases on development, highlighting their differential roles in biology despite their functional relationship and shared activities in cancer.

## 9. p53 Acetylation and Tumor Suppression

So, what is the role of p53 acetylation in tumor suppression by p53? The preponderance of data discussed in this review suggests it is essential since disruption of p53 acetylation generally results in loss of p53-mediated cell cycle arrest, senescence and/or apoptosis. Since its discovery, it has been widely accepted that p53 suppresses tumorigenesis through its transcriptional induction of one or more of those three key responses to cellular stress [1,2,13,14]. That prevailing view greatly influenced how researchers assessed p53 function over the years since nearly all studies of p53 acetylation (and other post-translational modifications) focused on how they affected p53 transcriptional control of cell cycle inhibitory and apoptotic genes, like *p21* and *Puma*. Indeed, one of the most compelling findings supporting a critical role for p53 acetylation in tumor suppression was the inability of an acetylation-deficient p53 mutant (8KR) to induce cell cycle arrest or apoptosis [40]. Yet recent work from the same group challenges the importance of p53 acetylation (at least at certain sites) and p53’s traditional functions (cell cycle arrest, senescence and apoptosis) to tumor suppression [261].

Specifically, Li *et al.* showed that mice defective for p53 acetylation at three sites (the 3KR mutant) retained the ability to suppress early-onset spontaneous tumorigenesis despite lacking p53-mediated cell cycle arrest, senescence and apoptosis [261]. The 3KR mouse p53 protein contains arginine mutations at K117, K161 and K162, analogous to a dual mutation of human p53 at K120 and K164. Mice bearing a single mutation at K117 (K120 in human p53), which are selectively defective in p53-dependent apoptosis, were likewise fully competent for inhibiting *de novo* tumor formation. These findings suggested that acetylation of those sites is dispensable for tumor suppression, at least for spontaneous thymic lymphoma. The role of other established sites of p53 acetylation, such as K320 and other C-terminal lysines, in p53-mediated tumor suppression still needs to be tested. Knock-in mice deficient in C-terminal acetylation (namely 6KR and 7KR mice) were generated but spontaneous tumorigenesis was not reported [43,44]. Moreover, the importance of p53 acetylation in stress-induced (e.g., radiation- or oncogene-mediated) tumorigenesis is a key unanswered question. Prior work suggests it may be essential for tumor suppression under those settings. That is because p53 acetylation is required for its canonical functions (cell arrest, senescence or apoptosis) following DNA damage and oncogene activation in cell lines, and retention of at least one of those functions is critical for tumor suppression in mice challenged with those stresses. For example, in a mouse model of irradiation-induced lymphoma, ARF-mediated p53-dependent senescence has been shown to be required for preventing tumor formation [262].

A key aspect of future studies will be determining the contribution of p53 acetylation to the regulation of its less conventional gene targets and how that relates to tumor suppression. In particular, emerging evidence suggests that p53 control of cellular metabolism and reactive oxygen species (ROS) levels may be critical for cancer prevention [13,14]. Li *et al.* found that the tumor suppressive 3KR mutant of p53 retains the ability to regulate genes (e.g., *TIGAR*, *GLS2* and *GLUT3*) that limit glucose uptake, glycolysis and ROS generation, implying those activities may be vital to tumor suppression [261]. Clearly, acetylation of the lysines mutated in p53-3KR is not required for regulating those metabolic genes but the involvement of other p53 acetylation events is not known. More recently, p53 transcriptional repression of malic enzymes and consequent NADPH production (critical for anabolic metabolism) was found to drive p53-mediated senescence and suppress the growth of xenograft tumors [263]. While these exciting findings are consistent with the paradigm that metabolic reprogramming promotes cancer [264], more studies are needed to test and verify the importance of metabolic gene control by p53 (as well as other non-traditional targets) to tumor suppression. In turn, studies exploring the role of p53 acetylation in regulating those genes and pathways should provide much needed insight into the significance of p53 acetylation to tumor suppression.

## 10. Conclusions

Acetylation of p53 is a complex process that involves many different proteins and multiple layers of regulation. Even after many years of research investigating the acetylation of p53 and its other post-translational modifications, many questions still remain. In response to different cellular signals, there is extensive crosstalk between acetylation and other p53 post-translational modifications, including phosphorylation, ubiquitylation, neddylation, sumoylation, and methylation. These result in transcriptional activation or repression of various p53 target genes that help the cell choose between life and death. Given the dispensability of p53-mediated cell cycle arrest and apoptosis to the prevention of spontaneous tumorigenesis [17,261], additional studies examining the impact of p53 acetylation on less studied gene targets and processes relevant to tumorigenesis, such as metabolism, are strongly warranted. Another important step in improving our understanding of p53 function will be clarifying how its acetylation and consequent biological outcomes are dictated by various types of cellular stress and/or the extent of cell damage. The analysis of knock-in mice expressing different acetylation-deficient forms of p53, as in the Li *et al*. study [261], that are challenged with different forms of stress (e.g., radiation or crosses with mice expressing activated oncogenes in various tissues) should be informative in that regard while also further testing the importance of p53 acetylation to tumor suppression.

In past and current studies, the use of p53 mutants with lysine-to-arginine substitutions has been helpful in teasing apart the mechanistic and biological roles of individual *vs.* combined sites of acetylation. Yet overexpression of p53 mutants in cells (where the stoichiometry of transcriptional complexes is aberrantly affected) has yielded different results than gene replacement studies in which the mutant allele is regulated under the endogenous promoter. For example, an overexpressed human p53-K120R/K164R mutant retains the ability to induce *p21* transcription and cell cycle arrest whereas an endogenously regulated, analogous mouse mutant (3KR; K117R/K161R/K162R) is defective for p53-dependent *p21* regulation, cell cycle arrest and senescence [40,261]. While both forms lack p53-mediated apoptotic gene control due to loss of K120 acetylation, the expression level of the mutant clearly influences its regulation of other target promoters, such as *p21*. Thus, caution must be exercised when interpreting typical p53 mutant overexpression and knockdown-replacement experiments *vs.* gene replacement studies.

Another experimental consideration worth noting as the field moves forward is cell context and the use of tumor derived cell lines (as most studies have done to date) as opposed to normal cells. One could argue that analyses performed in both settings have been valuable in determining the role of acetylation and other post-translational modifications in controlling p53 function. In transformed cells, the influence of post-translational modifications on p53 activation would be expected to resemble processes and outcomes occurring within a tumor (minus effects of the tumor microenvironment), reflecting events required for p53 to inhibit an existing cancer. In normal cells, either in a mouse or cultured primary cells, analyses should reveal the molecular events enabling p53 to maintain genomic integrity and prevent cellular transformation. All p53 regulatory mechanisms are intact in this context, consequently p53 post-translational modifications (where there is considerable redundancy) may have more of a “fine tuning” effect on its function, as suggested by p53 mutant knockin mice [43,44,75]. The impact of the same post-translational modifications on p53 activity and biological response may be different and more evident in neoplastic cells where one or more pathways that normally control p53 activity are dysregulated. As such, investigations of p53 regulation and activity in non-transformed cells may not “confirm” findings obtained in cancer cells but rather complement those studies by elucidating mechanisms under a distinct context. Both approaches, when carefully interpreted in light of possible cell type/species/context-specific roles of p53, as well as differential activity of endogenous *vs.* exogenously expressed p53, should provide meaningful insights into our understanding of p53 regulation and function.

Multiple anti-cancer therapies are being developed to reestablish active forms of p53 in tumors. Some of these mechanisms include directly re-expressing p53 in tumors by adenoviral delivery systems, blocking formation of Mdm2-p53 complexes (e.g., nutlins), reactivating mutant forms of p53 (e.g., via small peptides) or inhibiting the p53 deacetylases (e.g., tenovins) [28,29]. Targeting factors that promote or inhibit specific p53 post-translational modifications, which would then initiate distinct transcriptional programs, may provide an even more focused approach of treating cancer patients. For example, one could speculate that MOZ-induced acetylation of p53 at K120 and K382 is crucial for suppressing Ras-mediated tumorigenesis via induction of *p21* and cellular senescence [85]. Therefore, strategies that selectively promote MOZ-mediated p53 acetylation may be beneficial in patient tumors that express activated Ras and wild-type p53. Continued advances in understanding the role of acetylation in controlling p53 activity, in conjunction with its many other levels of regulation, will ideally lead to more personalized and efficient p53-based cancer therapy.
